# OPEN Hackathon at the TUM School of Medicine, Germany

**DOI:** 10.3205/zma001597

**Published:** 2023-04-17

**Authors:** Katharina Mosene, Celestine Kleinesper, Georg Prokop, Friedrich Caroli, Daniel Teufel, Pascal O. Berberat, Marjo Wijnen-Meijer

**Affiliations:** 1 Technical University of Munich, School of Medicine, TUM Medical Education Center, Munich, Germany; 2 University of Hamburg, School of Education, Hamburg, Germany

**Keywords:** curriculum development, Hackathon, co creation, student engagement, innovation lab

## Abstract

The OPEN Hackathon of the Technical University of Munich (TUM) 2020 set out to address challenges and potential solutions for medical education at the School of Medicine to kick off the 2020/21 winter semester. The event lasted 36 hours, during which medical students, teachers and staff members had the opportunity to tackle current problems in education and to develop co-created, customized solutions through creative teamwork for the School of Medicine at the TUM. The resulting solutions are now being realized and implemented in teaching. This paper describes the process and organization of the hackathon. Furthermore, the result of the evaluation of the event are described. In this paper, we aim to present the project as a valuable pioneer in the field of developing medical-educational topics within the framework of innovative methodological formats.

## Abstract (de)

Der TUM MEC OPEN Hackathon der Technischen Universität München (TUM) 2020 hat sich zum Auftakt des Wintersemesters 2020/21 mit Herausforderungen und Lösungsansätzen für die medizinische Ausbildung an der Medizinischen Fakultät beschäftigt. Während der 36-stündigen Veranstaltung hatten Medizinstudierende, Lehrende und Mitarbeitende der Fakultät die Möglichkeit, sich mit aktuellen Problemen in der Lehre auseinanderzusetzen und in kreativer Teamarbeit maßgeschneiderte Lösungen für die eigene Fakultät zu entwickeln. Die daraus resultierten Lösungen werden nun in die Lehre implementiert und strukturell umgesetzt. Dieser Beitrag beschreibt den Ablauf und die Organisation des Hackathons, außerdem werden die Ergebnisse der Evaluation der Veranstaltung diskutiert. Mit diesem Einblick wollen wir das Projekt als wertvollen Wegbereiter im Bereich der Erarbeitung von medizinisch-didaktischen Themen vorstellen, die im Rahmen innovativer methodischer Formate entwickelt werden.

## 1. Introduction

### 1.1. Background

Universities, as educational institutions, are committed to the ongoing development of their teaching and learning methods. Innovative educational formats allow students to intensively engage with subjects and help prepare them for future professional challenges, like cooperative work in diverse teams and communicative negotiation processes [1], [2], [3]. Using hackathons or idea competitions to address the school's need for innovation and expand the participants' soft skills therefore seems reasonable. A hackathon is a block event where ideas are generated and developed within a group. This solutions-based format originally from the computer sciences has since found great resonance in other disciplines as well; hackathons are being held at the government level (eg. [https://wirvsvirus.org]) as well as in academia [4].

Because of their high level of impact and ability to find solutions, hackathons were originally used primarily to develop products, yet they can also be adapted to other settings. They usually include a series of interrelated process steps. A hackathon opens with organizational preparation, followed by problem definition, recruitment of the group, development of a solution, presentation, and lastly evaluation. Because of that last step (judgement), hackathons can also be seen as competitions, while the process beforehand is more similar to conferences [5]. They generally match the following aspects:

1. **Short period of time**: the participants work closely together to solve a problem within a short period of time – usually a few hours or days [6].
2. **Winning award:** the participants' motivation is boosted through a reward. Usually, the best idea in a competition of visions is put into practice which motivates the participants to engage further [7].
3. **Group work:** most hackathons are voluntary; the participants either have an interest or an idea for a structural solution, or they want to join a team with a consisting idea. Teams are created from the very beginning and remain together until the end of the hackathon. This creates a learning situation where a high sense of community is created through intensive collaboration between the participants within the project groups. This type of group work was found to be highly productive and thus promises great opportunities for educational development [2].

In the past, several universities have held healthcare hackathons. However, these hackathons have primarily focused on product- and technology development, like the Stanford University's hackathon on June 5-6, 2016, where one of the winning teams developed the idea for a smartphone-based application to identify medication labels for the visually impaired [8]. The largest international association for medical education, AMEE, has also organized Hackathons several times [https://www.jchui.me/projects/ameehacks/]. Inspired by those models, the Medical Education Center (MEC) of the Technical University of Munich (TUM) and the student union developed the idea to improve the TUM School of medicine's own education within the frameworks of a hackathon.

Using the practical example of the TUM MEC OPEN Hackathon & Ideas Competition, this paper evaluates the various aspects to be considered when running such an event and provides an insight into how to profitably establish such formats within the area of educational development.

#### 1.2. Hackathon concepts and models 

An essential aspect of hackathons involves group dynamics with the well-known stages of the formation of teams: *forming, storming, norming, and performing* [9]. In the first step *(forming & storming)*, the group identifies the task or problem as well as the information and competencies needed to solve it. Hereby the group gets to know each other by sharing professional and personal experiences to assign subtasks appropriately. During the norming phase, the group members work on their subtasks and develop a fruitful way of working with each other. In the final stage, during the performing period, the group members work effectively in this mode of operation. 

A guideline by Cobham et al. [10] sets parameters for a successful Hackathon: a length of two to three days, an honorarium or equivalent compensation for the organizing team, a balance between a free-creative and competitive atmosphere, a broad range of diverse disciplines involved, and a realistic objective to stimulate self-efficacy among the participants.

An international evaluation in the field of medical innovation by Wang et al. [8] examined a total of eight Hackathons in China, Brazil, and the United States. The empirical survey focused on the learning progress of the 161 participants. The participants gave a standardized self-assessment before and after the hackathon so that their learning progress could be evaluated. The results were positive in all areas of evaluation (“perceived degree of learning”, including learning about Biodesign, lean startup methodology and prototyping a solution [8]), leading the authors of the study to conclude that hackathons are an effective method of medical didactics, especially because of the active involvement of the students structuring their own learning processes. However, there is a lack of large-scale studies and standards for the evaluation of hackathons, both in the field of medical education as well as in the field of educational development. 

Looking into the latest publications within the field of educational development through closely involving teachers and students there are some points, which underline the importance of event formats like hackathons. 

One important topic is the understanding of the huge important of co-creation to foster involvement and commitment because teachers and learners get a better understanding of mutual perspectives on education. This also leads to more positive, inclusive and democratic learning environment, increased internal motivation, and higher quality of the educational design [11]. These dynamics do not go without saying, fostering co-creating within curriculum development also always means reducing power differences, fostering openness, creating an atmosphere of shared responsibility and a motivation for collaborative problem solving. For hackathons this also means introducing feedback skills and a balanced communication culture, as well as support from staff members facilitating the co-creation process and “student-as-partner” conception [11], [12], [13]. Healey et al. in 2016 [14] stated that Curriculum design is probably the area where engagement through partnership (of teachers, faculty staff and students) is least well developed.

Those findings led us to finally implement a hackathon in the area of educational development at the TUM School of Medicine.

## 2. Project description

### 2.1. Project idea 

The idea for the project, which was funded by the *TUMs Ideas Competition 2020* [https://www.tum.de/studium/lehre/chancen-fuer-die-lehre/ideenwettbewerb/] was based on the successful Hackathon held by the *Hochschulforum Digitalisierung* [https://hochschulforumdigitalisierung.de/de/online-hackathon] and other project partners at the beginning of May 2020, in which various challenges of digital teaching & learning were addressed in a problem-solving competition involving various universities in Germany. Our Hackathon took this as a starting point and focused on the concrete challenges of our medical school environment. Our aim was to increase involvement of all stakeholders of university life (teachers, students and staff members) within educational development and to foster a long-term commitment to innovative teaching and learning settings in the various departments of our medical school. These objectives were also facilitated through the solution-oriented focus of the event, and the idea of incorporating the projects resulting from the hackathon into an Innovation lab. The Innovation Lab includes staff from the TUM MEC and two experienced medical students. In weekly meetings, they pushed the project forward and elaborated it through brainstorming processes. 

#### 2.2. Project development 

After the idea of the hackathon was born, it was developed and implemented by a team of employees and student assistants over a period of about four months. Together with the medical student union, weekly meetings were held to initiate the marketing of the hackathon via social media, plan the event and the cultural program, and press ahead with setting up the infrastructure for holding the hackathon. The topics addressed during the Hackathon were mostly submitted and developed by participating students and teachers (see Chapter 3 – Results: topics submitted by participants were “MedWoch”, “MindYourHealth”, “Mentoring”, “Healthy Climate“, “Error Culture”). There were also several topics selected by the TUM MEC, based on the results of the evaluation of the study program of the summer semester 2020, in order to identify educational settings and structures that could be improved (see 3.1 – Results: topics selected by the TUM MEC were “Digital Campus”, “Find IT!”, “HOW TO”). The TUM MEC, in cooperation with the Medical Student Union provided the organizational and technical (infra-)structure for the TUM MEC OPEN Hackathon.

##### How did you hear about the event?

**Figure 1:**
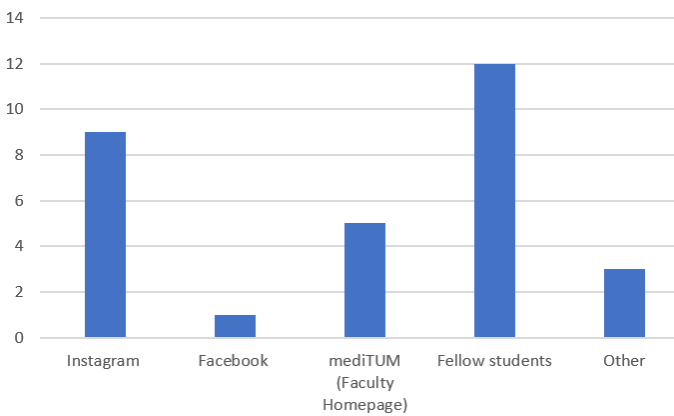
Ways that participants heard about the event

The participants were recruited through a variety of different media and mailing lists. Advertising was made among the teachers as well as among the students, though recommendations by colleagues or fellow students were the main reason for the large number of participants (see figure 1 (Fig. 1)).

**Figure 2:**
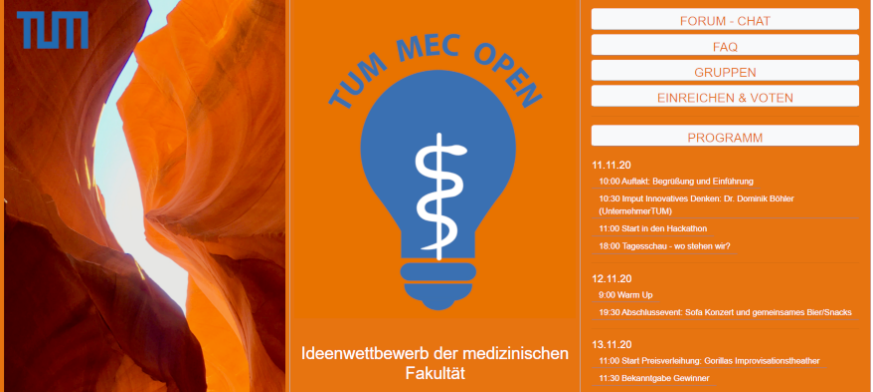
Design Moodle course – German version

Due to the pandemic, the Hackathon at the start of the winter semester 2020/21was an online event and mainly held as a Zoom conference. The groups were provided with additional tools for developing their ideas, such as Miro boards (online whiteboard) or mind-map tools, for collaborative work and for documentation. For each tool, detailed manuals were provided in advance, and the facilitators of the groups were also briefed on the objectives of the event. The complete program, including all groups and relevant links, was available in a Moodle course (course management system) set up for the competition (see figure 2 (Fig. 2)). 

Participants were thus able to join groups at any time and keep up to date with the latest developments. A Rocketchat (communication platform) was provided for global communication and data exchange. Experienced students and staff members of the TUM MEC chaired and guided the working groups, which met frequently to collaborate, based on the time capacities of the group members.

The 36h ran as follows: The participants of the hackathon were given a welcome speech to get them in the mood for the next 36 hours and were briefly familiarized with the schedule for the coming days. After that, the teams started with problem pitching and idea development. Trained moderators, qualified by the organization, with a view to group formation, collaboration and communication and TUM MEC team members were present in the respective groups to support the concrete elaboration of the topics during the working sessions. In the evening, the progress of the first day was summarized in a re-enacted daytime news broadcast. On the morning of the second day, an entrepreneurship workshop was held where participants learned idea development and teamwork skills. Afterwards, the groups continued working on idea development and developing their video pitches. On the evening of the 2nd day before the pitch deadline, there was a concert by a musician connected via Zoom, with the aim of providing some entertainment at the end of the 36 hours. Around noon of the following day, the jury selected the winners. The award ceremony was duly concluded with a digital performance by the improvisational theater, which also focused on and creatively framed the subject of educational development.

## 3. Results

### 3.1. Project results

Following the hashtag #36hoursfortheteaching (in German #36hfürdieLehre), the groups have developed several projects, which have been presented as video pitches within the framework of the contest and judged and honored by the jury, composed of the Dean of Studies, TUM MEC staff, teachers and students, and the crowd (all participants) and are now being successively implemented in teaching.

Here is an overview of the developed projects:

- **medWoch:** *Learning is through the stomach!* One of the winning projects, which is student-driven and based on social exchange between a small group of students and lecturers on a specific subject in an informal setting.
- **MindYourHealth: **A project driven by the student council that focuses on mental health and offers support in difficult situations.
- **Mentoring: **One of the winning projects. Driven by the student council, the Lab supports the structural establishment of a mentoring program at the medical school.
- **Healthy Climate – Climate Change in Teaching:** Another highly student-driven project, where the Innovation Lab supports the anchoring in classical teaching, i. e. in the form of a facultative additional Moodle course, a seminar and an incorporation into the cross-disciplinary core lectures.
- **Digital Campus:** The winning project of the crowd voting. This project aims at implementing a concise Moodle structure for the faculty's courses, mapped to the schedule, and including templates for lecturers to ensure a consistent design and to simplify the creation of digital courses.
- **Find IT!** The jury's award-winning project. Throughout the virtual and hybrid semester, the lack of accessibility to all the information has become evident. Therefore, a *Wiki for teaching* will be set up, mainly focusing on digital and innovative teaching approaches, allowing improved search for content as well as cross-linking.
- **Error culture – learning from mistakes: **This project aims at embedding educational material about teamwork and failure communication within the curriculum. With the support of the Lab, it will be introduced as a Summer School, as the topic is highly relevant and addresses a broad range of needs.
- **HOW TO** is a project of the Medical Training Center, focusing on the establishment of a hybrid training ground for medical skills and abilities. As far as this is to be offered both, virtually, but above all analogously, it is implemented in the winter semester 2021/2022 with the support of the Innovation Lab.

#### 3.2. Project evaluation

To measure the quality of the content, structural and social aspects, an evaluation was carried out among the participants. The evaluation data was collected using an online survey to allow the participants flexibility and to preserve anonymity. The construction of the questionnaire served to describe and evaluate concrete facts by the respondents [15]. When constructing the questionnaire, the focus was on wording and formulation of the questions in addition to the low effort required to complete the questionnaire. Closed questions were asked in order to ensure the comparability of the data; at the same time, open questions were asked in order to capture aspects of the evaluation that had not been considered [15].

A total of nine questions were asked, three of which were multiple choice questions about how the participants became aware of the ideas competition, what role they fulfilled, and which of the topics offered were most interesting. The fourth question addressed the perception of various organizational and social aspects and was formulated as a Likert scale with a five-fold gradation with the options "fully agree", "agree", "neutral", "agree less" and "disagree". This was followed by three open questions on what the participants would like to see in the next Hackathon, what was good and what was missing. The questionnaire ended with the question whether the participants would participate again. 

**Figure 3:**
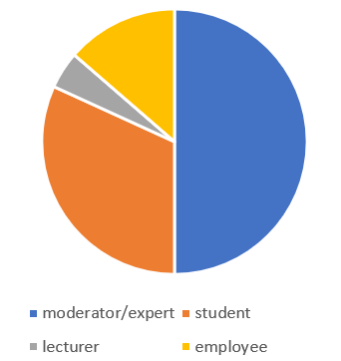
Role of the respondents during the Hackathon

The survey ran for a period of four weeks, during which participants of the Hackathon were invited by mail to participate in the online survey (see figure 3 (Fig. 3)). A total of 25 respondents took part in the online evaluation, thereof 12 teachers, 7 students and 3 employees of the university. As we had 60 participants, the response rate was 41.2%. Of the questionnaires returned, six were incomplete. These missings were considered since the questionnaires aim is to utilize every impression of the participants and the sample was very small [15].

The different backgrounds of the respondents are relevant for the interpretation of the results because the perception was made from different roles which partly overlap.

In general, the responds were quite consistent. Major differences were found in the area of organizational aspects of the hackathon; students preferred a short three-day-event, whereas employees and experts would have preferred to have more time. Overall, the respondents described the possibilities of hackathons being implemented into university life as largely positive. About 70% of the respondents state that they would like to participate in a hackathon again.

*Respectful interaction* in the cooperation between students and teachers was rated with 75 percentage points for the “fully agree” answer option without significant differences between different roles. 

With 62.5 percentage points, 15 respondents fully agree with the statement that the *use of the tools (Zoom, Miro, Moodle, Rocket Chat)* was useful.

The following exemplary free-text answers can be presented in the category “What was particularly good?”

- That the event took place digitally,
- the interplay between topic-related teamwork and a joint social program,
- intensive engagement with a topic,
- the use of tools and accessibility,
- the overall organization,
- food and entertainment were top notch and should be maintained,
- the Moodle Hackathon website was great and the hub of the hackathon.

Further free text fields gave the respondents the opportunity to express suggestions and proposals for improvement about the organization, content and process of the hackathon. In response to the question “What would you like to see more of at the next Hackathon?” nine respondents expressed ideas, below are examples of some of the responses:

- More people participating,
- early communication so that mentors which guided the groups can better prepare for the event,
- more experts should be involved.

Regarding the different projects, the evaluation results show that the category “Teaching and living error culture” was rated as the most interesting topic with 16.6 percentage points. This was followed by the categories “wiki for teaching”, “digital campus” and “climate change and health” with 12.5 percentage points each. 29.1 percent did not indicate a ranking of the most important topics.

All involved participants have practiced key generic skills like to identify problems and develop solutions in a results-oriented and sustainable manner, to work interdisciplinary and collaboratively in the virtual space, to develop and structure digital, innovative medical courses and to fill them with content, to apply basic techniques of communication, documentation, and visualization in the virtual space. They also have become familiar with eye-level partnership between students, teachers & faculty stuff and methods of co-creation. This includes the training of organizational, communicative and social soft skills as well as the experience of linking people who would otherwise never have formed an alliance in everyday life at university, even though they are facing similar challenges and are therefore well placed to exchange ideas and experiences.

## 4. Discussion & conclusion

Due to the relatively small sample of only 25 respondents, the findings must be interpreted with caution. However, the results offer a first insight into the possibilities of hackathons as a teaching/learning event for educational development and thus can be used as a basis for further research, especially large-scale-assessments and qualitative interviews for motivational aspects to participate in hackathons, development of social skills and impact on job orientation. 

Hackathons represent an innovative method for educational development through interdisciplinary and interprofessional exchange [16]. The advantages of Hackathons include network expansion as well as the expanding of the participants set of soft skills, team role finding, and co-creation methods also promote self-reflection [2], [17].

The evaluation of the use of hackathons as a university didactic teaching format, also in the area of educational development show to be predominantly positive.

Nevertheless, there is of course room for improvement. In future, the TUM MEC OPEN Hackathon should reach even broader groups of students and teachers, and the number of participants should be increased. Also, the timing of the Hackathon must be reflected critically. Due to the pandemic and the accompanying mandatory changes to established teaching formats, staff members and teachers had to cope with heavy demands at that time. As a result, some of them regarded the event as an additional expense rather than a helpful setting for the further development of their own teaching. In the future, we also want to enhance involvement through even greater flexibility regarding timing and more room for active exchange with colleagues and fellow students.

In the future, it would be conceivable to organize the hackathons primarily by student hands within the framework of the student union. Due to the preparatory work of the TUM MEC team and the students who planned and implemented the hackathon in 2020, it would be possible to draw on expertise and infrastructure in the future. This would save resources, especially money, and at the same time ensure the regular organization of a hackathon.

Thanks to the impact of this experience, we have decided to continue with this type of event on a regular basis. In the future, a regular, annual Hackathon will serve as a catalyst, and the associated Innovation Lab, which is dedicated to implement developed projects, as a safeguard of active and participative development of education. The structure of the Innovation Lab is essential to make the hackathon not only a "nice" event but also impactful and profitable – this of course also needs a lot of resources. People and funds must be provided to sustainably establish and drive the project suspended in the Innovation Lab.

The interplay of the hackathon and the Innovation Lab ensures a sustainable and future-proof culture of innovation for teaching, learning, and shared campus life, even and especially within the *new normal*.

## Competing interests

The authors declare that they have no competing interests.
